# Serum sBCMA in primary and secondary antibody deficiency

**DOI:** 10.1093/cei/uxaf065

**Published:** 2025-09-29

**Authors:** Danai Bagkou Dimakou, Nicholas E Peters, Siobhan O Burns, Alex G Richter, Adrian M Shields

**Affiliations:** Clinical Immunology Services, School of Infection, Inflammation and Immunology, College of Medicine and Health, University of Birmingham, Birmingham, UK; Clinical Immunology Services, School of Infection, Inflammation and Immunology, College of Medicine and Health, University of Birmingham, Birmingham, UK; Department of Clinical Immunology, University Hospitals Birmingham NHS Foundation Trust, Birmingham, UK; Institute of Immunity and Transplantation, University College London, London, UK; Department of Immunology, Royal Free London NHS Foundation Trust, London, UK; Clinical Immunology Services, School of Infection, Inflammation and Immunology, College of Medicine and Health, University of Birmingham, Birmingham, UK; Department of Clinical Immunology, University Hospitals Birmingham NHS Foundation Trust, Birmingham, UK; Clinical Immunology Services, School of Infection, Inflammation and Immunology, College of Medicine and Health, University of Birmingham, Birmingham, UK; Department of Clinical Immunology, University Hospitals Birmingham NHS Foundation Trust, Birmingham, UK

**Keywords:** common variable immunodeficiency, primary immunodeficiency, secondary immunodeficiency, vaccination, plasma cells, BCMA

## Abstract

B-cell maturation antigen (BCMA) is a B cell surface receptor that regulates B cell activation, proliferation and survival. BCMA can be cleaved from the cell surface, producing soluble BCMA (sBCMA), which has been studied as a disease biomarker in systemic lupus erythematosus, multiple sclerosis and multiple myeloma. Reduced sBCMA concentrations have been associated with the severity of different primary antibody deficiencies. We explored the relationship between sBCMA concentrations, humoral immune responses to SARS-CoV-2 vaccination and disease complications in 107 individuals with primary (PAD) and secondary antibody deficiency (SAD) enrolled in the COVID-19 in Antibody Deficiency (COV-AD) study. Serum sBCMA concentrations were significantly reduced in PAD compared to healthy controls and asymptomatic selective IgA deficiency. Individuals with X- linked agammaglobulinemia and common variable immunodeficiency (CVID) demonstrated the lowest serum concentrations of sBCMA. sBCMA concentrations in SAD were highly variable. Amongst individuals with CVID, peripheral blood CD19 count, but not sBCMA concentrations discriminated SARS-CoV-2 vaccine responders. sBCMA was significantly lower in individuals with CVID and bronchiectasis and outperformed serum IgA and IgM concentrations in discriminating this subgroup. sBCMA was not associated with any other complication of CVID. Our data highlights the potential of sBCMA as biomarker to support the assessment of antibody deficiency. In PAD, sBCMA may contribute to the risk stratification of disease severity and identify those at risk of bronchiectasis. In SAD, it may identify subgroups that would benefit from intensive monitoring and therapy.

## Introduction

B-cell maturation antigen (BCMA), also known as tumour necrosis factor receptor superfamily member 17 (TNFRSF17), is a cell surface receptor expressed throughout the B cell lineage, with heightened expression on activated B cells, plasmablasts, and plasma cells. BCMA is the surface receptor for B-cell activating factor (BAFF) and A proliferation-inducing ligand (APRIL); ligation of BCMA regulates B cell activation, proliferation and the survival of long- lived, antibody-producing plasma cells [1].

Membrane-associated BCMA can be cleaved by gamma-secretase [2], to produce soluble BCMA (sBCMA), a decoy receptor that counter-regulates the B cell axis [3]. sBCMA is detectable in human serum and cerebrospinal fluid and is a putative biomarker of disease activity in systemic lupus erythematosus and multiple sclerosis [2], and a tumour marker and therapeutic target in multiple myeloma [4]. Studies have also investigated whether serum sBCMA concentrations can discriminate different primary antibody deficiencies [5].

Maglione *et al.* and Guerra-Galan *et al.* have independently found reduced serum sBCMA concentrations differentiated severe antibody deficiencies from other milder conditions with high positive and negative predictive values [5, 6]. Herein, we expand on these findings by exploring the relationship between serum sBCMA concentrations, disease complications and *de novo* responsiveness to SARS-CoV-2 vaccination in different immune deficiencies.

## Methods and materials

The COV-AD study was a national UK study that enrolled 525 participants with antibody deficiency (defined as serum IgG <4 g/L receiving long-term antibiotic prophylaxis or any individual receiving immunoglobulin replacement therapy) between April 2021 and September 2022 to examine the immunological response to SARS-CoV-2 infection and vaccination [7] (REC reference 20/HRA/1817).

As part of ongoing mechanistic investigations, we undertook a 1500-aptamer proteomics screen (SomaScan, SomaLogic, Boulder, CO, USA), using serum samples from 113 COV-AD study participants taken 1–2 months following their second vaccine dose, followed by subgroup analyses based on underlying diagnosis, complications and vaccine responsiveness. Comparison of the serum proteome of individuals with common variable immunodeficiency (CVID) (*n* = 55) to those with secondary antibody deficiency (SAD) (*n* = 38) yielded 57 differentially abundant proteins in univariate analysis. Although no protein target remained statistically significant following correction for multiple comparisons, serum BCMA concentrations were found to be 1.86-fold higher in SAD compared to CVID (CVID—1813 relative fluorescent units (RFU) vs. SAD—3369 RFU, *P* = 0.003).

We explored this observation by measuring serum sBCMA concentrations by enzyme-linked immunosorbent assay (R&D systems, Human BCMA/TNFRSF17 DuoSet ELISA, 1/50 serum dilution) in 107 individuals from COV-AD, 23 individuals with asymptomatic selective IgA deficiency, 10 healthy controls and 6 individuals with monoclonal gammopathies of undetermined significance or multiple myeloma (paraproteinemia group) as positive controls.

Data were analysed using Graph Pad Prism 10.0 (GraphPad Software, San Diego, California, USA) using the statistical tests described in the figure legends.

## Results

Demographic parameters of the study and control cohorts and immunological characterisation of the COV-AD cohort are provided in Tables 1 and 2, respectively.

**Table 1.** Study cohorts demographics

	*N*	Age (yrs—median, iQR)	Sex (*n*, % male)	Primary sARS-CoV-2 vaccine course (dose 1 and 2) (*n*, %)		
Healthy controls	10	39 (30–53)	5 (50.0)	n/a		
Selective IgA deficiency	23	30 (19–45)	8 (34.7)	n/a		
^a^Paraprotein	6	74 (65–80)	2 (20.0)	n/a		
COV-AD cohort	107	56 (36–65)	38 (35.5)	*AstraZeneca ChAdOx1 nCoV-19* (*n* = 68/107, 63.6%)	*Pﬁzer BioNTech 162b2* (*n* = 38/107, 35.6%)	*Unknown* (*n* = 1, 0.8%)

^a^Isotypes in paraproteinaemia cohort: 1× free lambda, 2× IgM kappa, 1× IgM lambda, 1× IgD lambda, 1× IgG kappa (biclonal). One patient in the COV-AD cohort with genetically undefined common variable immune deficiency also had an IgA paraproteinaemia. No other individuals in the COV-AD cohort had detectable paraproteinaemia.

**Table 2.** Demographic and immunological characteristics of the COV-AD cohort

Condition	*N*	Age	% Male	Pre- treatment IgG (g/L)	IgA (g/L)	IgM (g/L)	CD19 B cell count (cells/10^9^/L)	Antibody response V2post (*n*, %)	Mean SARS-CoV-2 antibody response post V2 (anti-spike IgGAM ratio)	Receiving immunoglobulin replacement therapy (*n*, %)
Primary antibody deficiencies										
i. Predominantly antibody deficiencies										
Common variable immunodeficiency	49	52 (36–62)	46.9	2.97	0.33	0.45	0.19	26 (53.1%)	3.23	47 (95.9%)
Other primary antibody deficiencies	7	63 (52–76)	0.0	4.68	0.70	0.99	0.13	5 (71.4%)	5.58	7 (100%)
X-linked agammaglobulinemia	5	48 (24–55)	100.0	n/a	U	U	U	0 (0%)	n/a	5 (100%)
Specific polysaccharide antibody deficiency	3	46 (32–53)	0.0	8.76	1.15	0.57	0.12	3 (100%)	2.32	3 (100%)
ii. Combined immune deficiencies and other diseases of immune dysregulation										
Combined immunodeficiencies and other disease of immune dysregulation	6	26 (21–38)	33.3	3.19	1.24	0.74	0.07	5 (83.3%)	5.02	6 (100%)
Secondary antibody deficiencies										
Secondary antibody deficiency	37	61.0 (49–69)	21.6	3.24	0.73	0.75	0.45	26 (70.0%)	4.28	31 (83.8%)

Participants’ underlying immunological diagnosis was made according to the European Society of Immunodeficiency Clinical Working Party criteria. ‘Other primary antibody deficiency’ encompasses individuals who do not fulfil the diagnostic criteria for CVID, XLA, or any monogenic immunodeficiency but are still believed to have a primary humoral immunodeficiency. Amongst participants with a clinical diagnosis of common variable immune deficiency, six participants had a suspected monogenic cause accounting for their clinical phenotype: three individuals—heterozygous germline variants in cytotoxic T-lymphocyte-associated protein 4 (*CTLA4*), 1 individuals—heterozygous germline variant in nuclear factor NF-kappa-B p100 subunit 2 (*NFκB2)*, one individual—heterozygous germline variant in phosphatidylinositol-4,5-bisphosphate 3-kinase catalytic subunit delta isoform (*PIK3CD*) and one individuals—heterozygous germline variant in sterile alpha motif domain containing nine like (*SAMD9L*). Amongst individuals with a clinical diagnosis of combined immune deficiency, three individuals had a suspected monogenic cause accounting for their clinical presentation: one individual—heterozygous germline variant in signal transducer and activator of transcription 3 (*STAT3*) producing a hyper IgE phenotype, one individual—germline variant in forkhead box P3 (*FOXP3*) producing an immunodysregulation polyendocrinopathy enteropathy X-linked syndrome phenotype and one individual with a gain-of-function mutation in signal transducer and activator of transcription 1 (*STAT1*). Immunological data are presented as the mean value of the cohort. Antibody results are reported as a mean IgGAM ratio (optical density compared with calibrator) and results > 1.0 are defined as seropositive (full method described in [6]. U, undetectable/below limit of detection.

**Figure 1.**
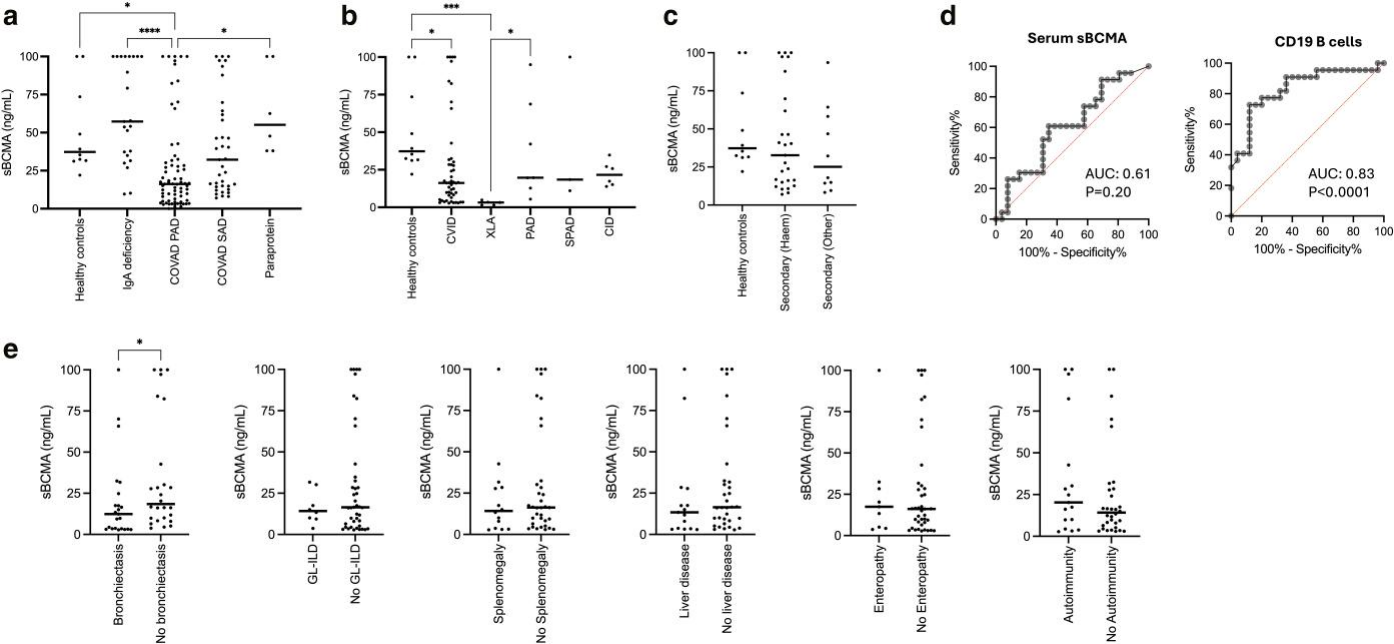
Serum sBCMA concentrations in (a) healthy controls and disease states, (b) different primary antibody deficiencies, and (c) secondary antibody deficiencies associated with haematological (Haem) and non-haematological disease (other). (d) Receiver operator characteristic curves demonstrating performance of serum sBCMA and peripheral blood CD19^+^ B cell count as biomarkers of a detectable humoral immune response following two doses of SARS-CoV-2 vaccine. (e) Comparison of sBCMA concentrations in individuals with CVID with and without organ specific or systemic complications. *—*P* < 0.05, ***—*P* < 0.0001 (Kruskall–Wallis Test with Dunn’s test for multiple comparisons (a-c) and Mann–Whitney test (e) performed)

Serum sBCMA levels were significantly lower in individuals with primary antibody deficiencies compared to individuals with selective IgA deficiency and healthy controls (median serum sBCMA concentration: primary antibody deficiency: 16.3 ng/ml vs. selective IgA deficiency: 57.3 ng/ml, *P* < 0.0001 vs. healthy controls: 37.2 ng/ml, *P* = 0.03, Kruskal–Wallis test with Dunn’s multiple comparisons test) (Fig. 1a). Across different primary antibody deficiencies, individuals with XLA had the lowest concentrations of sBCMA (Fig. 1b). The median serum concentration of sBCMA in individuals with CVID and XLA were both significantly lower than healthy controls. Although median sBCMA levels in less severe primary antibody deficiencies and specific polysaccharide antibody deficiencies were lower than healthy controls, this was not statistically significant. A sBCMA of >30.87 ng/ml discriminated between individuals with CVID and those with asymptomatic selective IgA deficiency with sensitivity 82.61% (CI 62.86% to 93.02%) and specificity 75.51% (CI 61.91% to 85.40%). A wide range of serum sBCMA concentrations were observed amongst individuals with SAD (Fig. 1a); no significant differences were observed between the SAD cohort, individuals with primary antibody deficiencies and healthy controls, nor within the SAD group depending on its cause (Fig. 1c).

53.1% (*n* = 26/49) of individuals with CVID mounted a measurable serological response to two doses of SARS-CoV-2 vaccination. No significant differences in sBCMA concentrations were observed between vaccine responders and non-responders with CVID (median sBCMA CVID responders: 16.6 ng/ml vs CVID non-responders 11.8 ng/ml, *P* = 0.20). Peripheral blood CD19^+^ B cell count (AUC 0.83) performed significantly better than sBCMA (AUC 0.61) as a biomarker of vaccine non-response in CVID (Fig. 1d—comparison of receiver operator characteristic curves: z-statistic 2.21, *P* = 0.03). A peripheral B cell count of <0.139 × 10^9^/L was associated with non-response to SARS-CoV-2 vaccines with 90.9% sensitivity and 64.0% specificity, in keeping with SARS-CoV-2 vaccination being a *de novo* antigenic challenge when vaccines were deployed in the UK in early 2021. Combining serum sBCMA concentrations and peripheral CD19^+^ count did not improve the receiver operator characteristic curve. sBCMA concentrations also did not discriminate between SARS-CoV-2 vaccine responders and non-responders in the broader primary antibody deficiency cohort, excluding individuals with XLA who do not make humoral immune responses to vaccination.

**Figure 2.**
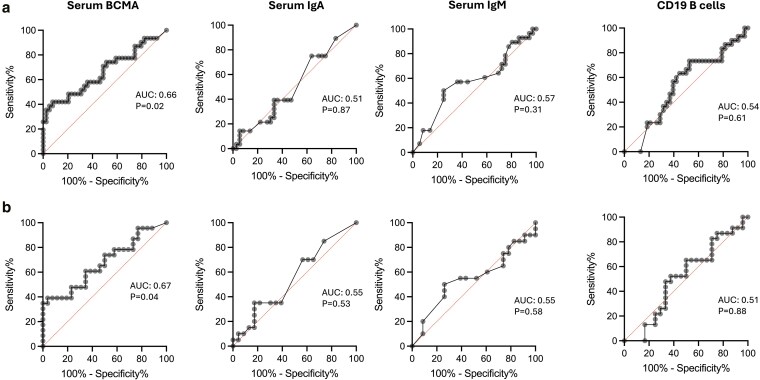
Biomarker performance in discriminating individuals with primary antibody deficiency (a) and common variable immunodeficiency (b) with and without bronchiectasis. Serum IgA and serum IgM concentrations were not available for three participants with and without bronchiectasis, respectively

In individuals with CVID, those with bronchiectasis had significantly lower concentrations of serum sBCMA (median concentration 12.4 ng/ml vs. 18.5 ng/ml, *P* = 0.04) than those without bronchiectasis. Serum sBCMA concentrations did not associate with any other complications of immune dysregulation in CVID (Fig. 1e). Amongst individuals with CVID, and more broadly amongst those with primary antibody deficiency, serum sBCMA concentrations, but not serum IgA, IgM concentrations or peripheral blood CD19^+^ B cell count, showed relatively limited potential to discriminate between individuals with and without bronchiectasis (Fig. 2a and b).

For example, sBCMA concentration <15.6 ng/ml distinguished individuals with CVID and bronchiectasis from those with CVID without bronchiectasis with 60.9% sensitivity and 65.4% specificity. In 30 individuals with CVID with known pre-treatment, IgG concentrations, there was no correlation between pre-treatment IgG and sBCMA concentrations (*r* = 0.13, *P* = 0.49), but positive correlations were observed between serum IgA concentrations (*r* = 0.72, *P* < 0.0001) and serum IgM concentrations (*r* = 0.55, *P* = 0.002).

## Discussion

Our study demonstrates serum sBCMA is reduced in individuals with PAD, particularly in those with XLA and CVID, supporting the hypothesis that serum sBCMA is a biomarker of the severity of antibody deficiency. Our findings are broadly concordant with those of Maglione *et al.* and Guerra-Galan *et al.* who also found individuals with XLA and CVID had reduced serum BCMA concentrations [5, 6]. We found the performance of sBCMA in discriminating CVID from sIgAD was comparable to Guerra-Galan *et al.* (sensitivity 88.9%, specificity 87.0%, cut-off 15 ng/ml) and Maglione *et al.* (sensitivity 73%, specificity 96%, cut-off 15 ng/ml) although our study identified a higher cut-off of 30.87 ng/ml. The absolute difference between the cut-off values is modest—a 1.5% difference within a 2-log dynamic assay range—and likely arises from minor methodological differences between the studies. In SAD, the observation that 32% of individuals had sBCMA lower than the median sBCMA concentration of individuals with CVID, suggests sBCMA may have utility in identifying individuals with SAD who require more intensive monitoring or therapeutic intervention.

We provide evidence that sBCMA may be a useful adjunct to immunoglobulin concentrations, B cell enumeration and vaccine responsiveness when characterising immunity in individuals with antibody deficiency. In individuals with CVID, we observed positive correlations between contemporaneously measured serum IgA, IgM, and sBCMA. Long-lived plasma cells are the major source of serum IgA, and to a lesser extent IgM. These correlations support the hypothesis that sBCMA is a biomarker of residual plasma cell numbers. In contrast, we did not observe a correlation between IgG concentrations at diagnosis and contemporaneous sBCMA concentrations. The ongoing loss and/or failure to replenish IgG producing plasma cells over time likely confound such a direct comparison. Our data are largely concordant with Guerra-Galan *et al.* who reported a negative correlation between the VISUAL score and sBCMA levels [7]. The VISUAL score [8] incorporates vaccine response data, IgA and IgM concentrations, CD4 enumeration and switched memory B cell proportions at diagnosis into a numerical score that facilitates identification of individuals with CVID who may develop a more severe phenotype. Guerra-Galan’s *et al.* did not examine the relationships between the individual components of the VISUAL score and sBCMA and the net contributions of each component to the observed relationship is uncertain. Although Maglione *et al.* [5] did not report significant associations between immunoglobulin concentrations and sBCMA in a heterogeneous cohort of individuals with primary antibody deficiencies, the CVID subgroup was not explored.

*De novo* humoral immune responses to vaccination (e.g. following SARS-CoV-2 vaccination early in the COVID-19 pandemic), arise from the activation and differentiation of naïve CD19^+^ B cells and their survival as antigen-specific antibody secreting cells. In contrast, serum sBCMA mainly derives from long-lived plasma cells. With an estimated half-life of 24–36 h, sBCMA concentrations act as a surrogate of the current total plasma cell pool and the humoral immune memory it contains. In combination, serological response to vaccination and sBCMA evaluate different phases and compartments of the B cell axis. Individuals with XLA, who have complete deficiency of the B cell lineage and are unable to make any humoral responses have the lowest median sBCMA concentrations. In contrast, we have found variable vaccine responsiveness [7] and serum sBCMA in antibody deficiencies where either (i) the B cell lineage remains partially intact, allowing replenishment of the plasma cell pool, or (ii) proximal defects in the B cell lineage emerge after some long-lived humoral immunity has been established. Future studies may consider the relationships between vaccine responsiveness, sBCMA, and infection burden in primary and secondary antibody deficiencies.

Bronchiectasis is a known long-term complication of antibody deficiency: individuals with XLA have a 75% risk of developing bronchiectasis by 43.5 years of age and have the lowest levels of sBCMA in the disease groups we studied [9]. We found that individuals with CVID and known bronchiectasis have significantly lower levels of sBCMA than those without this complication. Previous studies, based on patients enrolled in the UK PIN registry, found individuals with PAD who had reduced concentrations of both IgA (<0.8 g/L) and IgM (<0.5 g/L) were more significantly more likely to have a diagnosis of bronchiectasis, compared to those with normal serum concentrations of these immunoglobulin isotypes [10]. In our study, only sBCMA demonstrated any potential as a biomarker that could discriminate between those with and without bronchiectasis. This may be due the high prevalence of more severe antibody deficiency in the COV-AD cohort; amongst patients with CVID enrolled in COV-AD, 88% were deficient in IgA (median concentration 0.2 g/L), 76% deficient in IgM (median concentration 0.3 g/L) and 67% deficient in both IgA and IgM as defined by the criteria used within the UK PIN registry paper. For the wider PAD cohort, 77% were IgA deficient (median concentration 0.1 g/L), 70% were IgM deficiency (median concentration 0.3 g/L), and 63% deficient in both.

Future cross-sectional studies may examine the relationship between the severity of bronchiectasis and serum biomarkers including sBCMA and immunoglobulin concentrations, while longitudinal studies may consider whether those with low sBCMA are at risk of developing bronchiectasis, thereby enabling earlier preventative measures.

## Data Availability

The authors confirm the data supporting the findings of this study are available within the article. Requests for the raw data should be directed to the corresponding author and will be considered by the COV-AD study steering group upon reasonable request.
